# Barriers and Considerations for Diagnosing Rare Diseases in Indigenous Populations

**DOI:** 10.3389/fped.2020.579924

**Published:** 2020-12-14

**Authors:** Carla S. D'Angelo, Azure Hermes, Christopher R. McMaster, Elissa Prichep, Étienne Richer, Francois H. van der Westhuizen, Gabriela M. Repetto, Gong Mengchun, Helen Malherbe, Juergen K. V. Reichardt, Laura Arbour, Maui Hudson, Kelly du Plessis, Melissa Haendel, Phillip Wilcox, Sally Ann Lynch, Shamir Rind, Simon Easteal, Xavier Estivill, Yarlalu Thomas, Gareth Baynam

**Affiliations:** ^1^IRDiRC Scientific Secretariat, National Institute for Health and Medical Research, Paris, France; ^2^National Centre for Indigenous Genomics, Australian National University, Canberra, ACT, Australia; ^3^Department of Pharmacology, Dalhousie University, Halifax, NS, Canada; ^4^Precision Medicine, Platform on Shaping the Future of Health and Healthcare, World Economic Forum, San Francisco, CA, United States; ^5^Institute of Genetics, Canadian Institutes of Health Research, Government of Canada, Ottawa, ON, Canada; ^6^Human Metabolomics, North-West University, Potchefstroom, South Africa; ^7^Facultad de Medicina, Center for Genetics and Genomics, Clinica Alemana Universidad del Desarrollo, Santiago, Chile; ^8^Institute of Health Management, Southern Medical University, Guangdong, China; ^9^KwaZulu-Natal Research Innovation and Sequencing Platform, University of KwaZulu-Natal, Durban, South Africa; ^10^Rare Diseases South Africa, Johannesburg, South Africa; ^11^Australian Institute of Tropical Health and Medicine, James Cook University, Smithfield, QLD, Australia; ^12^Department of Medical Genetics, University of British Columbia, Victoria, BC, Canada; ^13^Faculty of Maori and Indigenous Studies, University of Waikato, Hamilton, New Zealand; ^14^Oregon Clinical and Translational Research Institute, Oregon Health and Science University, Portland, OR, United States; ^15^Department of Mathematics and Statistics, University of Otago, Dunedin, New Zealand; ^16^National Rare Disease Office, Mater Misericordiae University Hospital, Dublin, Ireland; ^17^Academic Centre on Rare Diseases, University College Dublin, Dublin, Ireland; ^18^Western Australian Register of Developmental Anomalies, Perth, WA, Australia; ^19^Quantitative Genomics Laboratories (qgenomics), Esplugues de Llobregat, Barcelona, Spain; ^20^Genetic Services of Western Australia, Department of Health, Government of Western Australia, Perth, WA, Australia; ^21^Faculty of Health and Medicine, Division of Pediatrics, University of Western Australia, Perth, WA, Australia; ^22^Telethon Kids Institute, University of Western Australia, Perth, WA, Australia; ^23^Faculty of Medicine, University of Notre Dame, Fremantle, WA, Australia; ^24^Faculty of Science and Engineering, Spatial Sciences, Curtin University, Perth, WA, Australia; ^25^Faculty of Medicine, Notre Dame University, Perth, WA, Australia; ^26^School of Population and Global Health, University of Melbourne, Melbourne, VIC, Australia

**Keywords:** Indigenous populations, genomics, diagnosis, rare diseases, equity

## Abstract

Advances in omics and specifically genomic technologies are increasingly transforming rare disease diagnosis. However, the benefits of these advances are disproportionately experienced within and between populations, with Indigenous populations frequently experiencing diagnostic and therapeutic inequities. The International Rare Disease Research Consortium (IRDiRC) multi-stakeholder partnership has been advancing toward the vision of all people living with a rare disease receiving an accurate diagnosis, care, and available therapy within 1 year of coming to medical attention. In order to further progress toward this vision, IRDiRC has created a taskforce to explore the access barriers to diagnosis of rare genetic diseases faced by Indigenous peoples, with a view of developing recommendations to overcome them. Herein, we provide an overview of the state of play of current barriers and considerations identified by the taskforce, to further stimulate awareness of these issues and the passage toward solutions. We focus on analyzing barriers to accessing genetic services, participating in genomic research, and other aspects such as concerns about data sharing, the handling of biospecimens, and the importance of capacity building.

## Introduction

The number of people worldwide living with a rare disease is estimated between 263 and 446 million (1), and there are an estimated 370–500 million Indigenous peoples in the world, spread across 90 countries and speaking the major share of the world's almost 7,000 languages (2). People with rare diseases frequently suffer from inequitable access to diagnosis and treatment, and the majority of the world's population is currently outside the perimeter of readily accessible diagnostic applications.

Indigenous peoples represent a rich diversity of cultures, religions, traditions, languages, and histories. Globally, the health status of Indigenous peoples frequently varies significantly from that of non-Indigenous people population. While making up <5% of the world's population, Indigenous people account for 15% of the poorest (3). Indigenous people life expectancy is up to 20 years lower than that of the non-Indigenous population (3). Children born into Indigenous families often live in remote areas where there may be relative underinvestment in, or access to, basic social and medical services. Indigenous families, be they metropolitan or more remote, may have limited or no access to culturally appropriate care, due to linguistic, geographic, financial, and other factors.

It takes an average of 5 years to diagnose a child with a rare disease (4). Given known inequities in Indigenous health care, and clinical experience of rare disease diagnosis in Indigenous families (5), the diagnostic odyssey is even more challenging for Indigenous persons living with rare diseases, and there is a need for research assessing the impact of this on the Indigenous communities. These delays often severely limit the benefits of an accurate diagnosis for Indigenous peoples, including psychological relief, reduced isolation, reduction of unnecessary investigations, access to improved or best practice medical care, genetic counseling, clarification of recurrence risk, provision of additional reproductive options, and in some cases access to social and educational services (6).

Over the last 20 years, Indigenous people's rights have been increasingly recognized through the adoption of international instruments and mechanisms, such as the United Nations Declaration on the Rights of Indigenous Peoples (UNDRIP) in 2007 (7), the American Declaration on the Rights of Indigenous Peoples in 2016, 23 ratifications of the Indigenous and Tribal Peoples Convention from 1991, the establishment of the United Nations Permanent Forum on Indigenous Issues (UNPFII), the Expert Mechanism on the Rights of Indigenous Peoples (EMRIP), and the UN Special Rapporteur on the Rights of Indigenous Peoples (UNSR) (8). There is also an increasing awareness and formal recognition of the human rights of people living with rare, including undiagnosed, diseases (9). Collectively, these factors provide a convergent tapestry of rights and recognition to support advancing rare diseases diagnosis for Indigenous people.

Significant diagnostic delays and inequitable access to diagnostic services for people living with a rare disease has prompted a response by initiatives at local, national, and international levels. By way of just one example, the US National Institutes of Health (NIH) established in 2008 the Undiagnosed Diseases Program (UDP) (10), and subsequently, the Undiagnosed Disease Network (UDN; https://undiagnosed.hms.harvard.edu), and similar programs were established globally by countries participating in the Undiagnosed Diseases Network International (UDNI; http://www.udninternational.org) (11). Such programs are advancing the integration of genomics and other “omics” technologies into medical practice to reduce diagnostic delays and improving patient and family journeys. Increasing equity, including that for Indigenous peoples, is a key consideration for this and other diagnostic initiatives.

Increased diagnostic certainty may be provided through various means such as clinical expert opinion and clinical consensus including with application of diagnostic criteria where they exist (for both genetic and non-genetic rare diseases); through other phenotypic approaches such as facial analysis and other imaging; and through the building of Indigenous genomic databases and the identification of rare pathogenic variants in Indigenous Peoples with a suspected rare genetic disorder, or other “omics” technologies (e.g., epigenetic signatures). The International Rare Disease Research Consortium (IRDiRC; www.irdirc.org) has set the goal of enabling all people living with a rare disease to receive an accurate diagnosis within 1 year of coming to medical attention (12). However, significant barriers remain regarding access to diagnosis for Indigenous populations, including but not limited to poorer access to (gen)omic technologies and the research that drives them, which prevent Indigenous peoples from receiving appropriate benefits from (gen)omic and other new knowledge. Hence, IRDiRC has formed a taskforce to identify the barriers for the diagnosis of rare genetic diseases in Indigenous peoples, and areas in which there are opportunities for improvement. This is adding to the knowledge base informing future development of recommendations for improving diagnosis of genetic and rare diseases for Indigenous peoples. In this article, we focus our analysis specifically on the limited access to diagnostic services and clinical expertise, underrepresentation in genomic databases and genomics research in general, concerns with the handling of biospecimens, data use and data sharing, and the importance of building capacity within communities. We provide some examples of initiatives addressing these barriers, which are briefly summarized in Table 1.

**Table 1:** Initiatives to improve Indigenous access to genetic and genomic research and health care.

**Initiative**	**Country**	**Description**	**Area**
Lyfe Languages (38)	Australia	Initiative to translate the standardized description of phenotypic abnormality in man (the Human Phenotype Ontology) into Indigenous languages.	Indigenous languages initiative
Better Indigenous Genetics (BIG) Health Services project (42)	Australia	National partnership project that aims to improve the provision of clinical genetic services to Aboriginal and Torres Strait Islander people.	Indigenous clinical genetic service
National Center For Indigenous Genomics (48)	Australia	Body within the Australian National University (ANU) dedicated to bringing the benefits of genomic medicine to Indigenous Australians by creating Indigenous genomic data resources.	Indigenous genomics database
Silent Genomes (49)	Canada	Precision medicine project which aims to create a database of background genetic variations for Indigenous populations living in Canada and globally.	Indigenous genomics database
Aotearoa Variome (51)	New Zealand	The Aotearoa Variome project will assemble genomic resources and develop a catalog of genetic variants present in the genomes of New Zealanders.	Indigenous genomics database
A Framework for Enhancing Ethical Genomic Research with Indigenous Communities (60)	International	Ethical framework informed by community-based participatory research (CBPR) principles, to engage Indigenous people and communities in genomic research.	Indigenous ethical frameworks
Te Mata Ira: Guidelines for Genomic Research with Maori (61)	New Zealand	It outlines a framework for addressing Māori ethical issues within the context of genetic or genomic research.	Indigenous ethical frameworks
He Tangata Kei Tua: Guidelines for Biobanking with Maori (62)	New Zealand	It describes a model for biobanks to guide culturally informed governance, operational, and community engagement activities.	Indigenous ethical frameworks
Ethical Conduct in Research with Aboriginal and Torres Strait Islander Peoples and Communities (69)	Australia	It provides values and principles to ensure that research conducted with or for Aboriginal and Torres Strait Islander people and communities, or their data or biological samples, is ethically conducted.	Indigenous ethical frameworks
Guidelines for Genomic Research with Aboriginal and Torres Strait Islander Peoples of Queensland (70)	Australia (Queensland)	Guidelines for researchers on how to engage and partner with Queensland communities for potential genomic-research projects.	Indigenous research guideline
Tri-Council Policy Statement 2: Ethical Conduct for Research Involving Humans (TCPS2) (71)	Canada	Chapter 9 outlines a framework for the ethical conduct of research involving Indigenous peoples.	Indigenous research guideline
Maori Data Sovereignty Network (Te Mana Raraunga) (79)	New Zealand	National-level Indigenous data sovereignty network. These networks are creating principles for Indigenous data sovereignty and Indigenous data governance to inform the appropriate management and sharing of data.	Indigenous data network
United States Indigenous Data Sovereignty Network (80)	United States	National-level Indigenous data sovereignty network.	Indigenous data network
Maiam nayri Wingara Aboriginal and Torres Strait Islander Data Sovereignty Collective (81)	Australia	National-level Indigenous data sovereignty network.	Indigenous data network
Research Data Alliance International Indigenous Data Sovereignty Interest Group (82)	International	Interest Group focus on building an international Indigenous Data Sovereignty research platform.	Indigenous data infrastructure
Global Indigenous Data Alliance (83)	International	International Indigenous Data Sovereignty Network advocating for rights and interests to data.	Indigenous data alliance
Care Principles of Indigenous Data Governance (84)	International	International framework for ethical use of Indigenous data.	Indigenous data framework
Genetic Education for Native Americans (89)	United States	Native-specific educational program on genetics.	Indigenous education
Summer Internship for Indigenous Peoples in Genomics (102)	International	Workshop training for Indigenous researchers in genomics.	Indigenous capacity building

### Access to Clinical Genetic Services

Clinical genetic service providers are at the front line for rare disease diagnostics and frequently act as the portal to and from translational research. Indigenous access challenges to these services, and associated testing, can include lack of referral and/or referral bias, location, cost, availability, and cultural appropriateness. This includes culturally appropriate education materials that resonate with Indigenous narratives and incorporate indigenous language; meeting places and clinical processes that accommodate for familial and community participation; consent processes adaptable to family structures; involvement of culturally appropriate support and liaison persons for addressing racism and discrimination; and cultural competency among genetic health-care staff and capacity building in genetic and rare diseases (be they genetic or environmental) in the primary care workforce. Each of these issues affects engagement in, and delivery of, rare disease diagnosis.

The total burden of rare diseases in Indigenous communities is unknown. Estimates of prevalence of rare disease in “mainstream” (i.e., predominantly non-indigenous ethnicity/ancestry) communities from the Orphanet database range from 3.5 to 5.9%, of which 71.9% have a hereditary basis (1). Assuming similar prevalence in Indigenous communities, this equates to 12–20 million indigenous people worldwide affected by at least one rare disease, based on an estimated worldwide population of 476 million indigenous peoples (8). While many Indigenous people live in metropolitan areas, some Indigenous groups are remote or nomadic with limited geographic access to genetic health care services—and therefore to a rare disease diagnosis. As an example, approximately 1 in 5 Indigenous Australians (Aboriginal and Torres Strait Islander peoples) live in remote areas and about 1 in 3 live in major cities (13). In Canada, half of Indigenous peoples (First Nations, Inuit, and Métis) live in a rural environment (14). For less developed populations globally, even though a doubling of urbanization occurred over the past 50 years, ~1 in 2 people still live in rural areas (15). Telehealth and outreach clinics are undoubtedly an important part of the matrix of approaches to accommodate geographic access issues. Moreover, there are other challenges for Indigenous families, in terms of technology, internet access, bandwidth, reliability, and cultural appropriateness. In the African context, the needs of people living with rare diseases must also be balanced with basic needs, such as nutrition and communicable-disease prevention (16).

While many rare diseases affect Indigenous populations at similar rates to non-indigenous peoples, some rare diseases are geographically and/or culturally concentrated and this has implications for tailoring research and clinical care. An example is that the Machado-Joseph disease (MJD)—also called spinocerebellar ataxia Type 3 (SCA3)—is estimated to be more prevalent among Aboriginal people from small communities in remote northern Australia than anywhere else in the world (17). Geographical isolation and cultural preference for large, closely tied families have resulted in disproportionately high numbers of people living with this condition. Currently, over 650 Aboriginal Australians are thought to be “at-risk” of developing the disease. The Australian MJD Foundation (MJDF; https://mjd.org.au) was established in 2008, as a community driven response to families affected by MJD, and has pioneered an innovative model of disability service delivery to meet the needs of Aboriginal communities in remote Northern Australia (17). Core to its principles is the implementation of a “two-way,” bicultural working model, i.e., the services are delivered in community by an Aboriginal community worker and a non-Aboriginal health or community service professional. The approach allows first language support which minimizes the potential for misunderstanding and disengagement for cultural mistakes. Another example from Canada is work with Gitxsan Health Society to identify the cause of sudden death, often at a young age, in this First Nation's community in northern British Columbia (18, 19). This resulted in the discovery of a mutation in the *KCNQ1* gene, which causes the Long QT syndrome and has now been found in ~1.5% of the community's population. The team worked with the community to ensure that the research was performed in a culturally safe manner. Long QT syndrome is a rare heart rhythm condition which can be lethal if left untreated, but with appropriate treatment and monitoring patients can live a normal life. Today, anyone with a predisposition to Long QT syndrome receives evidence-based care by a team of northern British Columbia health-care workers supported by the British Columbia Inherited Arrhythmia Program. An African example is glutaric aciduria type 1 (GA1), a rare disease that can be detected by newborn screening and is treatable. The Black South African population, including Indigenous (San and KhoeKhoe) and non-Indigenous peoples, has a predicted prevalence of this disorder that is 20 times higher (1:5184) than global average (1:100,000) (20).

Understanding the total burden of rare diseases in Indigenous populations within the country they reside, the identification of the actual conditions themselves and their individual prevalence and community distribution are significant knowledge gaps to be addressed in terms of tailoring access, and provision of, genetic health care. In addition to diagnostic and epidemiological initiatives, rare disease codification with granular and interoperable systems, such as Orphanet coding (21), in health data sets will be critical, as well as documentation therein of Indigenous status.

Financial access issues are also critical; these may be of particular relevance since poverty is more common among Indigenous, compared to non-Indigenous, people. Also, it is important that funding/reimbursement is sufficient to cover all aspects of diagnostic care, such as pretest and posttest culturally appropriate counseling—and not just the cost of a genetic test.

Given that Indigenous people are more likely to live regionally or remotely when compared to non-Indigenous people, methods to improve access to testing, service, and research will require a combination of approaches that embrace a mixture of telehealth, local capacity building, and outreach models. Approaches that map access closer to home and incorporate cultural, geographic, weather, and language factors will also be beneficial (e.g., Mappa; https://mappanews.org.au).

Poor access to clinical expertise at the point of care and lack of referrals (or referral bias) to genetics specialists is another barrier to diagnosis. Given the diversity and rarity of these disorders, diagnosis of rare diseases is often difficult. Rare in presentation, they can sometimes go unrecognized by practitioners. Moreover, expertise and specialist knowledge may be scarce (22). The development of the European Reference Networks (ERNs) by the European Commission in 2017 was a priority action to compensate for the scarcity of knowledge on rare diseases and improve access to clinical expertise across Europe (23). Preliminary data from the Australian Northern Territory Genetic Service suggested that rates of referral for Indigenous patients were less than half of what would be expected based on the population (24). This may be due to general lack of awareness of indicators of rare diseases by referring doctors, attributing symptoms to other more common disorders, or a belief that services are not accessible or will not be attended.

Recognizing a patient with a rare disease can be particularly challenging in remote areas where expertise in rare diseases does not exist and for ethnic minorities such as the Irish Traveler population, a distinct Irish ethnic minority. The Irish Travelers are an endogamous nomadic ethnic minority group with a very high incidence of rare diseases. As they practice inter-cousin marriage, a greater incidence of autosomal recessive genetic disorders is reported with many rare diseases occurring in this population with a prevalence threshold >5 in 10,000 (25–27). Clinicians who are less familiar with this population had until recently no resources to support more timely diagnosis of the rare disorders in Irish Traveler patients. A catalog of the disease mutations of known genetic disorders found among this ethnic group is now publicly available (26), and a database of genetic disorders for Irish Traveler is under development to facilitate diagnosis at the point of care (27). National and ethnic specific mutation databases that may be used, for example, to facilitate the provision of genetic services, have been developed for a number of populations worldwide. Some examples include the Mediterranean Founder Mutation Database (28), the Omani National Genetic Database (29), the Israeli National Genetic Database (30), the Moroccan Genetic Disorders Database (31), and the Amish, Mennonite, and Hutterite Genetic Disorder Database (32).

A lack of cultural appropriateness may also challenge Indigenous rare disease diagnosis. This may be due to language barriers, location and setup of clinical rooms, a lack of accounting for gender roles and responsibilities, or lack of awareness of other cultural norms and responsibilities. Communication between Indigenous patients and health-care practitioners' professionals continues to be problematic and is arguably the greatest barrier to the delivery of successful health care and translational research for Indigenous peoples (33). Communication barriers include language issues and the use of medical jargon. These undermine constructive practitioner–patient relationships and result in Indigenous patients and families feeling alienated, non-compliant, and disengaged from health care. Indigenous patients and families want to be informed about health and disease, while information is frequently lacking, inadequate, or presented in ways that are incongruent with indigenous peoples' beliefs, narratives, and experiences (34, 35). Communication issues are compounded when families are uninvolved in communication processes (36, 37). Additionally, a history of negative health-care experiences that parallel other negative colonization experiences results in mistrust and suspicion by Indigenous people of health practitioners and patient information provided (36). Building trust through sustained community partnerships and the use of culturally appropriate language and concepts are critical. One initiative to build the lexicon, narratives and trust to address rare diseases communication issues is Lyfe Languages (www.lyfelanguages.com) (38), which involves translating the standardized description of phenotypic abnormality in man (the Human Phenotype Ontology) into Indigenous languages through community and transgenerational partnerships. Launched with a focus on Aboriginal Australian languages, this has now extended to African and Swedish (Sami) languages. Where Indigenous people may be fluent in English, have English as a second, third, or fourth language, or speak a blended mixture of English and Indigenous languages, such as Aboriginal-English for example, outputs of Lyfe Languages have been adapted to these contexts. Recognizing that some Indigenous people may have no or intermittent internet access, Lyfe Languages has commenced the development of resources (e.g., Apps) that are variably web-based or mobile applications and designed to run with periodic syncing to a web platform, while also supporting scalable and bespoke hard copy (paper) resource development.

While there is a relative dearth of initiatives to improve Indigenous access to genetic and genomic care, from a non-disease-specific basis and an Australian perspective, over the last decade, a number of state-based, multi-state, and more recently national approaches in Australia have begun to address inequitable access to genetic diagnostics and genetic health care across the breadth of rare diseases. These both complement and learn from the extensive work of the MJD Foundation in Australia's Northern Territory. They include initiatives from The National Center for Indigenous Genomics (described further below); the work of the Western Australia (WA) Health Department, the Aboriginal Health Council of WA, and WA Aboriginal Communities (39), ACTG—Aboriginal Community-led Translation of Genomics (40); the work of Genetic Health Queensland and Queensland Aboriginal and Torres Strait Islander Controlled Health Services (41); the Better Indigenous Genetics (BIG) Project, a translational research project for improving genetic health care service delivery in Australia (42); and more recently the work of the POCHE Center for Indigenous Health (43). Each has specific foci and overlaps.

### Underrepresentation in Genomic Research and Reference Databases

The emergence of large-scale databases and biobanks, prompted by the proliferation of large-scale population genomics projects worldwide, has paved the way to genomic medicine. Globally, however, Indigenous peoples remain understudied and underrepresented in genome reference databases (44). For example, a recent analysis revealed that as of 2018, only 22% of individuals in genome-wide association studies (GWAS) were of non-European ancestry (45). People of African and Latin American descent and Indigenous people combined represented <4% of participants. Indigenous participants represented 0.02%, which equates to an alarming 250-fold underrepresentation based on the number of Indigenous people. This under-representation of ethnically diverse populations in human genomic studies has important implications for the interpretability of genomic variants and diagnostic assessments (46). As genome sequencing is increasingly used in the diagnosis of rare and undiagnosed conditions (47), reference genomes from more ethnically diverse populations are needed for reliable interpretation of results and ultimately the effective and responsible implementation of precision medicine in minority populations (16, 46).

A number of initiatives are seeking to address the gap in the availability of Indigenous genome reference databases and ensure culturally appropriate access to data by engaging and co-designing with Indigenous communities, ascertaining the data through trusted partnerships and making it available through mechanisms that embrace both FAIR and CARE principles, discussed further in section Cultural Considerations for the Use of Biospecimens and Data Sharing. The National Center for Indigenous Genomics (NCIG, https://ncig.anu.edu.au) is creating Australia's first database of Indigenous genomes for use in research of benefit to Indigenous Australians (48). The resource is being developed from an existing collection of Indigenous biospecimens held by the Australian National University (ANU) since the 1960s and supplemented by the ongoing addition of new material. Another Australian initiative includes the work of the Telethon Kids Institute to deliver the first whole-exome genomic reference data, and related data access processes, initiating with Western Desert People of Western Australia (40). In Canada, the Silent Genomes project launched in 2018 with the goals of reducing health-care disparities and improving diagnostic success for Indigenous children with genetic diseases plans the development of a database of background genetic variations for Indigenous populations from across Canada (49, 50). In Aotearoa/New Zealand, a similar initiative has commenced, with the goal of developing a catalog of genetic variants within Indigenous Māori, to expedite identification of causative variants and disease diagnoses, as well as development of gene-based diagnostics (51). Like the Silent Genomes project, this is co-led by Indigenous researchers and guided by Indigenous ethical frameworks relevant to the study populations (50).

Other efforts to include minority populations in human genetic studies include the Human Heredity and Health in Africa Initiative (H3Africa; https://h3africa.org), jointly funded by the NIH in the US and the Wellcome Trust in the UK to advance African-led genome research (52), the UK MRC-funded International Center for Genomic Medicine in Neuromuscular Diseases (ICGNMD) (53), and the NIH All of Us research program (https://allofus.nih.gov), a precision medicine initiative which plans to collect DNA and health data from one million people including American Indians and Alaska Natives (54). China has also launched the precision medicine initiative (55), supporting the cohort studies covering healthy population and special disease populations in different geographical areas, where the ratio of Han ethnicity and minority peoples varies among each other. One of the cohort studies with the funding from the precision medicine initiative, covering the registry for over 100 rare diseases, has been performed since 2016 and it recruited patients with a breadth of ethnicities in China (56).

However, aiming for a more diverse population representation in genomic research is not without challenges. In the US, for example, while efforts to recruit tribal nations in the All of Us cohort study are ongoing, a recent report from the Tribal Collaboration Working Group has underlined a range of issues that need to be addressed if tribes are to participate, including a need to recognize tribal sovereignty, rights, and interests (57). The report also raised questions about responsible data use, cultural appropriateness, biospecimen storage and access, and protection and benefits. In another study examining Indigenous Canadians' concerns for contributing their data to a genomic database, issues were raised around lack of trust due to historical transgressions, control and ownership of samples, privacy, and fear of discrimination (58).

Whereas experiences with unethical research practices have caused some individuals to mistrust health research and resulted in Indigenous peoples' hesitancy to participate in genomic research (59), guidelines and ethical frameworks for respectfully engaging with Indigenous communities have emerged in recent years to help build trust and repair relationship. Guidelines for genomics research with Indigenous peoples developed by countries such as the US, Canada, New Zealand, and Australia now provide broad protections for research participants, including protection for sample collection, secondary uses, benefits, and the right to withdrawal from research (59). A framework for enhancing ethical genomic research with Indigenous communities provides a set of principles for conducting respectful engagement, such as understanding tribal sovereignty and research regulation, engaging with the community throughout the research process, building cultural competency of researchers, improving transparency of research practices, building tribal capacity in the research process, and disseminating research findings in a community-accessible format (60). Additionally, the development of culturally appropriate guidelines such as *Te Mata Ira: Guidelines for Genomic Research with Maori* in New Zealand (61) provides directions on the cultural concepts, logic, and values that underpin Maori perspectives on genomic research and outlines a framework to engage communities in a culturally acceptable manner. A culturally informed biobank model that draws on Maori-informed practices has also been developed and provides a framework for considering cultural values in relation to Indigenous peoples in other countries, and society in general, to ensure that biobanking policies, procedures, and practices are culturally acceptable (62).

### Cultural Considerations for the Use of Biospecimens and Data Sharing

Indigenous cultural practices add complexity to the ethical issues surrounding genetic and genomic research, at both the individual and collective levels. Challenges to incorporate local cultural practices into research may negatively influence decisions about participation in a research project (63–65). For instance, indigenous perceptions that blood specimens and other tissue samples remain intimately connected to their ancestors and their lands raise ethical concerns related to the collection, storage, and use of biospecimens (63, 65). It also raises questions about collective ownership, tribal belonging, repatriation, and the use of biospecimens beyond national borders (63). In Australia, the NCIG addresses these challenges by including an Indigenous Majority Board on its governance, which is responsible for the custodianship of biological samples and for its management and use (66). Recently, the NCIG started to offer participants and relatives of deceased people whose historical samples are in its collection the option of having their remains repatriated to their place of origin (67). Research approaches that promote community participation, such as the application of community-based participatory research (CBPR) principles (68), the adoption of ethical research guidelines (61, 62, 69, 70), and the development of culturally informed policies (71, 72) offer ways to ensure that research involving indigenous peoples is premised on respectful relationships.

Indigenous concerns around data use and sharing is another barrier to research participation (73, 74). This is increasingly important as the current move toward broad data sharing and open data impacts the data governance rights of Indigenous nations (75). As a response, stricter mechanisms of control over biological samples and associated data are emerging by Indigenous Peoples' (e.g., tribal review/oversight boards, research codes, policies), and a global call for “Indigenous data sovereignty”—the right of Indigenous peoples and tribes to govern the collection, ownership, and application of their own data—is growing in intensity and scope (75, 76). Yet, at the same time, it was also acknowledged that stricter mechanisms of control to protect Indigenous rights may deepen the “genomic divide” by excluding them from the potential benefits of data sharing (77). This is especially relevant to rare diseases, where knowledge and expertise are limited and patient populations are geographically dispersed (78). Disparate data is a key barrier to detect rare diseases and the sharing of genomic and clinical data across country borders is imperative to help accelerate the time it takes to diagnose a rare disease. A counterpoint is that solutions that accommodate Indigenous concerns over data management and sovereignty and, through that build trust, may ultimately facilitate the scaling of data sharing for Indigenous and non-Indigenous people.

The reclaiming of Indigenous data sovereignty (IDS) entails Indigenous people being in control of their data and comprises the entitlement to Indigenous data governance (75). As the first Indigenous-governed genome facility in the world, the NCIG in Australia gives control of decision-making back to Indigenous people (66). The Canadian's Silent Genomes is also working in concert with Indigenous Canadian organizations and communities to establish processes for Indigenous-led governance of biological samples and genomic data (49). Additionally, growing IDS networks are working to shape open data principles to better respect the rights of Indigenous peoples, e.g., the establishment of national Data Sovereignty networks in New Zealand (Te Mana Raraunga; https://www.temanararaunga.maori.nz) (79), US (USIDSN; https://usindigenousdata.org) (80), and Australia (Maiamnayri Wingara; https://www.maiamnayriwingara.org) (81); the International Indigenous Data Sovereignty Interest Group at the Research Data Alliance (RDA IDS Group; rd-alliance.org) (82); and the Global Indigenous Data Alliance (GIDA; https://www.gida-global.org) (83). Released under the auspices of RDA, the CARE Principles of Indigenous Data Governance (collective benefit, authority to control, responsibility, ethics) provide the first international framework for the ethical use of Indigenous data (84). The Principles reflect the crucial role of data sharing and complement the existing FAIR (Findable, Accessible, Interoperable, Reusable) principles (85) as a key principle guiding equity and ethics in data open movements.

The need for phenotypic reference data parallels the need for genomic reference data (86). This will require dedicated initiatives for instance for imaging data, such as (3D) facial analysis (87) that can accommodate the same considerations as for (gen)omic data.

### Capacity Building and Empowerment

Engagement and consultation with community members, respect for cultural differences, and transparency around research are key elements for ethical research involving Indigenous peoples (60). These are seen as important steps toward forming research relationships built on trust, respect, and mutual interests, which in turn can enhance research participation. To this end, it is critically important to increase capacity among Indigenous and non-Indigenous peoples alike. Ensuring that non-Indigenous researchers have adequate cultural competency to engage with Indigenous communities in a research project often requires training before its onset. Funding bodies, such as the Australia's National Health and Medical Research Council (NHMRC) and Human Research Ethics Committees (HRECs), require researchers to demonstrate capacity to effectively conduct research with Aboriginal and Torres Strait Islander peoples (70). Recently, Silent Genomes published a document to support and foster cultural safety practices among genetic counselors working with Indigenous families (88). It is also currently developing educational materials to increase communities' genetic literacy (49). Promoting genomic literacy among Indigenous people and communities is an important area to enhance informed decision-making. The Genetic Education for Native Americans (GENA) pioneered such efforts by offering Native Americans workshops on genetics at national conferences. Today, GENA has developed into an educational program to Native Americans on genetic science (89). Although not targeted to Indigenous peoples, the NIH National Human Genome Research Institute (NHGRI; https://www.genome.gov) has developed tools and provides a list of useful educational resources to advance genomic literacy for the general public (90).

The current and projected shortage of medical geneticists and genetic counselors, who historically have provided the majority of genetic services, has created a need for genomic education of health-care professionals across disciplines (91, 92). The African Genomic Medicine Training (AGMT) Initiative, for example, was established to develop knowledge and skills of non-specialist providers in genomic medicine (93). Indigenous people, especially those living in rural and remote areas, have poor access to health services (94) and little to no access to genetic health services. An important aspect for addressing racial and ethnic health-care disparities and improving access to genetic services for underserved patients, and cross-cultural communication, is ensuring a more diverse genetic workforce (95). In a recent survey on the demographics of certified Genetic Counselors in the US, 90% of respondents identified themselves as Caucasian, while only 1% of respondents identified as black or African American. Just over 2% of respondents identified as Hispanic, 0.4% identified as American Indian or Alaskan Native (96). When compared to the racial/ethnic distribution in medical education and the health-care workforce, Black or African American and American Indian or Alaska Natives are also underrepresented (97). The Minority Genetic Professionals Network (https://minoritygenetics.org) was formed in 2018 as an initial step to develop a more diverse workforce of medical genetic professionals to meet the needs of racial and ethnic minority populations (98). In Australia, the Better Indigenous Genetics (BIG) Project will develop training courses targeted to build capacity of Aboriginal health care workers to collaborate in the provision of genetic health service and primary care for Indigenous people. Songlines and weaving are an educational initiative being developed as a partnership between the Western Australian Register of Developmental Anomalies, Genetic Services of Western Australia, and the Roy Hill Community Foundation to develop culturally appropriate, culturally endorsed, and culturally resonant education information for use in clinical genetic practice (99). The first educational product uses the Aboriginal Song Line narrative to explain basic concepts of genetics, inheritance, and genetic health care. Capacity building in Indigenous rare diseases policy will also be required.

The dynamics of the lack of racial and ethnic diversity in genomics is paralleled in the US science and engineering workforce as according to the most recent data, American Indians or Alaska Natives are underrepresented as are blacks and Hispanics relative to their presence in the overall US workforce (100). Diversification of the research workforce is one way to ensure that genomic research better serves the needs of diverse and under-represented communities (101). Furthermore, the inclusion of Indigenous researchers in genomics can bring greater transparency to the research process, facilitate the dialogue between researchers/universities and Indigenous communities, and foster the development of research skills in genomics within communities, thereby strengthening inclusivity in biomedical research (60, 102). The Summer internship for Indigenous peoples in Genomics (SING; https://www.singconsortium.org) program offers a successful model for training and retaining Indigenous researchers in genomics. Launched first in the US and further expanded to Canada, Aotearoa/New Zealand (www.singaotearoa.net), and most recently Australia, SING is a week-long workshop that aims to train Indigenous students, scholars, community members, and researchers with next-generation genomic and bioinformatics analyses and build capacity for scientific research involving Indigenous communities. SING operates internationally through the expanding network of the SING Consortium and has organized earlier this year the first international conference on Indigenous genomics (103). Capacity building in genomic science for Indigenous communities is also being supported through programs and translational activities associated with Silent Genomes and the Aotearoa Variome (50).

## Conclusion

Herein, we outline some challenges and barriers to rare disease diagnosis in Indigenous people, and some examples of initiatives raising awareness and advancing solutions to the related challenges to diagnostic equity. Serving the unmet needs of Indigenous people with undiagnosed rare diseases will require a multifarious and internationally coordinated response that is grounded in engagement, co-design, and trust. Ultimately, improving Indigenous rare disease diagnosis will reduce inequity and deliver discovery, innovation, and solutions that will benefit all.

## Author Contributions

GB conceived the study with input from all authors. CD'A wrote the manuscript in consultation with GB. All authors provided critical revision and approved the final manuscript.

## Conflict of Interest

The authors declare that the research was conducted in the absence of any commercial or financial relationships that could be construed as a potential conflict of interest.
